# A Combined Deep-Learning and Lattice Boltzmann Model for Segmentation of the Hippocampus in MRI

**DOI:** 10.3390/s20133628

**Published:** 2020-06-28

**Authors:** Yingqian Liu, Zhuangzhi Yan

**Affiliations:** 1 School of Communication and Information Engineering, Shanghai University, Shanghai 200444, China; zzyan@shu.edu.cn; 2 School of Electrical Engineering, Binzhou University, Binzhou 256600, China

**Keywords:** deep belief network, shape prior, lattice Boltzmann method, hippocampus segmentation

## Abstract

Segmentation of the hippocampus (HC) in magnetic resonance imaging (MRI) is an essential step for diagnosis and monitoring of several clinical situations such as Alzheimer’s disease (AD), schizophrenia and epilepsy. Automatic segmentation of HC structures is challenging due to their small volume, complex shape, low contrast and discontinuous boundaries. The active contour model (ACM) with a statistical shape prior is robust. However, it is difficult to build a shape prior that is general enough to cover all possible shapes of the HC and that suffers the problems of complicated registration of the shape prior and the target object and of low efficiency. In this paper, we propose a semi-automatic model that combines a deep belief network (DBN) and the lattice Boltzmann (LB) method for the segmentation of HC. The training process of DBN consists of unsupervised bottom-up training and supervised training of a top restricted Boltzmann machine (RBM). Given an input image, the trained DBN is utilized to infer the patient-specific shape prior of the HC. The specific shape prior is not only used to determine the initial contour, but is also introduced into the LB model as part of the external force to refine the segmentation. We used a subset of OASIS-1 as the training set and the preliminary release of EADC-ADNI as the testing set. The segmentation results of our method have good correlation and consistency with the manual segmentation results.

## 1. Introduction

The shape and volume of the hippocampus (HC) is altered in cases of Alzheimer’s disease (AD), schizophrenia and epilepsy, among other conditions [1]. Atrophy of the HC has been shown to be one of the first observable characteristics for the detection of AD or mild cognitive impairment (MCI) [2]. To diagnose the above diseases, the HC volume should be easily obtainable and reliably and consistently measured by MRI. However, the intensity distribution of different brain structures shows considerable overlap [3]. Not all edges are visible for HC, the white matter inferior to the HC is not always well resolved and a large part of its border with the amygdala is usually invisible.

Hippocampal segmentation methods include but are not limited to image-based methods, active contour models (ACM) [4], active appearance and shape models [5], atlas models [6] and deep learning methods [7]. Image-based and ACM methods suffer from a low robustness and accuracy and require extensive user interaction. Model-based methods such as active appearance and shape models can overcome the problems with previous methods and reduce user interaction at the expense of a large training set to build a general model. However, it is difficult to build a model that is general enough to cover all possible shapes of the HC. Atlas methods, especially multi-atlas based methods, have the advantage of enabling segmentation in individuals with great anatomical variability. The disadvantage of this kind of method is that it requires many registration operations, which increases its computational cost. Recently, deep learning methods have been applied in medical image segmentation, but the lack of golden standards for medical image segmentation limits the development of such methods. To solve this problem, the literature [8] uses the segmentation result of FreeSurfer [9] as the training label of deep convolutional neural networks.

The active contour model with prior information (usually shape and appearance) embedded into the external term [10,11] is robust and can solve the problem of boundary leakage. However, these shape and appearance priors are usually generated based on linear combinations of training shapes. The selection of training labels must consider the shape variability of the object to be segmented. Label alignment is a necessary step in building shape prior. In the process of segmentation, compared with the shape prior, the target to be segmented in the image may have different sizes and orientations, and it is also necessary to align the shape prior with the target to be segmented; that is, the target to be segmented is a similar transformation of the shape prior. However, it is less effective in handling nonlinear transformations like partial stretching and bending. Using ACMs with these priors, it is hard to model all variations present in the visual object of interest and the align process is time consuming. Therefore, we hope to find a patient-specific method to model the shape of the object to be segmented with a small number of training samples. Restricted Boltzmann machines (RBM) [12] is a graphical model with a layer of visible units and a layer of hidden units, where connections exist between the two layers but not between the units within each layer. This restriction facilitates inference with the model. As a generative model, RBM has been used to model brain tumor [13] and lung shape [14]. Considering that RBM lacks the ability to capture the global properties of complex shapes, some researchers used deep belief networks (DBN) to capture the global shape prior of left ventricle endocardium [15,16]. DBNs are composed of several RBM and can effectively model shape using limited training samples [17,18]. ACM with prior information from DBN can reduce the need of using a highly complex network structure.

However, a common drawback of the traditional solution methods for ACM is poor efficiency, rendering its utilization for time-critical applications problematic. Recently, the lattice Boltzmann (LB) method has attracted the attention of researchers for its natural parallel characteristics and clear physical meaning [19]. The partial differential equation (PDE) for image processing can be constructed from the LB evolution equation. From this perspective, the LB method can be regarded as an alternative solution method for PDE with higher efficiency. On the other hand, the application of the LB method in image processing can be regarded as a diffusion process; we will explain this viewpoint in Section 2.3. While LB methods have been successfully applied to image denoising [20], inpainting [21] and segmentation [22,23], there is little research on medical image segmentation. As far as we know, these studies include: Segmentation of tumors in 3D ultrasound images [24]; using the LB algorithm to solve the distance regularization level set (DRLS) to complete the segmentation of intracranial giant aneurysm thrombus [25,26]; embedding local statistical information into the LB external force term to segment brain white matter [27]; using the principal component analysis (PCA) method to extract the main components of HC labels as the shape prior of the LB segmentation model [28]. However, using PCA to obtain the shape prior is limited by the assumption that the shape prior is Gaussian distribution. This method also needs alignment and similarity transformation between the shape prior and the object to be segmented. Thus the method proposed in [28] has good segmentation results when segmenting the HC, which has little difference with the training samples, but the error is large when segmenting the HC, which has serious atrophy. In this work, we propose a DBN driven LB model for HC segmentation.

Using limited training samples, DBN can model shapes of HC. The combination of DBN and LB is patient specific and can get accurate segmentation results without a complex network structure. The shape prior inferred from DBN can solve the problem of boundary leakage caused by an ambiguous boundary, while the initial contour can reduce iterations for segmentation. Compared with the statistical shape prior, our method does not need label alignment and shape prior registration with the object to be segmented. The segmentation accuracy of the DBN-LB_joint is higher than using the DBN and LB methods independently, and it is higher than the PCA-LB_joint and the cRBM [29] -LB_joint, and is comparable with state of the art methods.

## 2. Method

### 2.1. Method Overview

**Figure 1:**
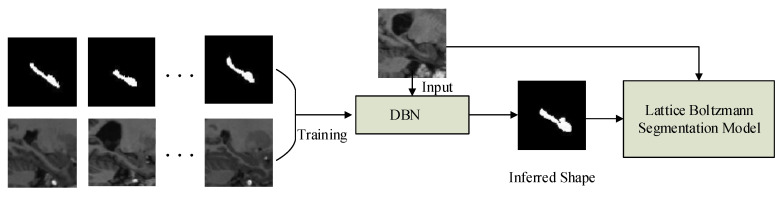
Block diagram of the developed algorithm.

The block diagram of the proposed method is depicted in Figure 1. The method is carried out in two stages: (i) The shape of the HC is inferred by using the trained DBN; (ii) the inferred shape is used for initialization and also is incorporated into the LB model for segmentation. The DBN is trained during an offline training process to obtain its optimum values of parameters. After training, we deploy the system to perform the automatic segmentation task. The two stages and the LB method are further elaborated as follows.

### 2.2. Shape Inferring

**Figure 2:**
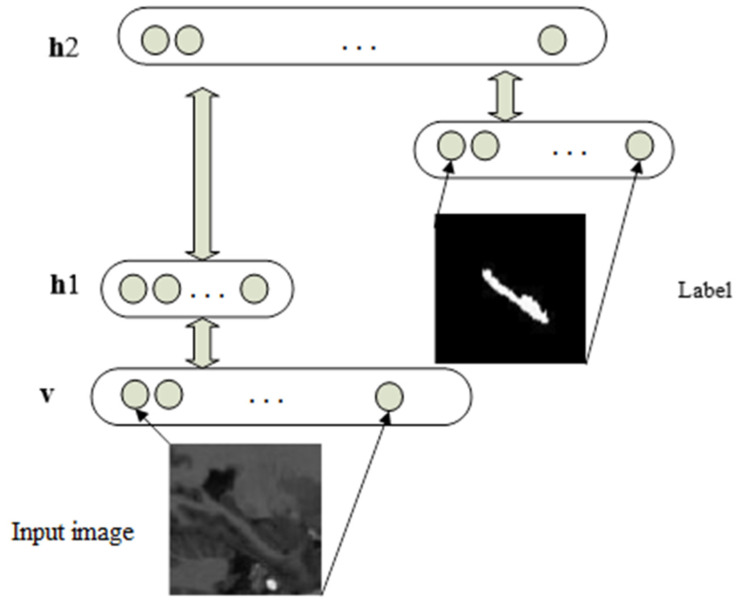
The deep belief network (DBN) used for shape inferring.

We utilize and train a DBN as depicted in Figure 2 to infer the shape of the HC. We exploit the model with the following joint probability:

(1) $$P(v,h_{1},h_{2},L)=P(h_{2},h_{1},L)P(h_{1}|v),$$

where v is a vector representation of the input image, L∈(0,1) represents the label of v, h denotes the hidden variables, and $-\log P(h_{2},h_{1},L)\propto \varepsilon_{\mathrm{RBM}}(h_{2},h_{1},L)$ with

(2) $$\varepsilon_{\mathrm{RBM}}(h_{2},h_{1},L)=b_{2}^{Τ}h_{2}-a_{1}^{Τ}h_{1}-a_{L}^{Τ}L-{(h_{2})}^{Τ}W_{2}h_{1}-{\left(h_{2}\right)}^{Τ}W_{L}L,$$

representing the energy function of RBM. $b_{2}$, $a_{1}$, $a_{L}$ are the bias vectors; $W_{2}$ and $W_{L}$ are weight matrices. Moreover, we have

(3) $$P(h_{2}|h_{1})=\underset{j}{\Pi }P(h_{2}(j)=1|h_{1})=\sigma (b_{2}(j)+h_{1}^{Τ}W_{2}(:,j)),$$

with $P(h_{1}(j)=1|v)=\sigma (b_{1}(j)+v^{Τ}W_{1}(:,j))$, where $\sigma (x)=\frac{1}{1+e^{-x}}$, the operator (j) returns the jth vector value and (:,j) returns the jth matrix column.

This DBN is trained layer by layer in an unsupervised way by stacking RBMs. The error minimized during this unsupervised training is the reconstruction error of the visible input. The result obtained from the previous RBM is used as “visible” input for the next RBM. The supervised training begins only at the top RBM, when the segmentation label L is provided as visible inputs. The inference process consists of taking the input image and performing bottom-up inferences, until reaching the top two layers, and then initializing the layer L = 0 and performing Gibbs sampling on the layers $h_{2}$, $h_{1}$, and L until convergence.

### 2.3. Explanation of Lattice Boltzmann Method

**Figure 3:**
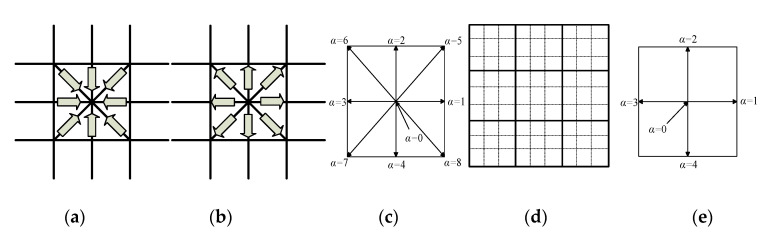
Explanation of the lattice Boltzmann (LB) method for image processing. (**a**) Collision process; (**b**) streaming process; (**c**) D2Q9 structure; (**d**) sub-pixel denoted by dotted box and pixel denoted by solid box; (**e**) D2Q5 structure.

LB methods are usually composed of two steps: Collision and streaming steps. The collision step is shown in Figure 3a: Particle collisions happen and then velocities of particles change; as a result particle density functions are redistributed in each grid. As shown in Figure 3b, the streaming step means the moving of particles after the collision step. The LB evolution equation in image processing is redefined in [19]:

(4) $$I_{\alpha }(r+e_{\alpha }\Delta t,t+\Delta t)-I_{\alpha }(r,t)=\frac{1}{\tau (I)}\left[I_{\alpha }^{eq}(r,t)-I_{\alpha }(r,t)\right]+\Delta t\cdot F_{\alpha },$$

where I(r,t) denote the gray level of a pixel r=(x,y) at time *t*; it is treated as the mass of sub-pixels. In Figure 3d, the square in the solid box represents a pixel, and the square in the dotted box represents a sub-pixel. $I_{\alpha }(r,t)$ is the gray distribution function on the sub-pixel in direction $e_{\alpha }$. $I_{\alpha }^{eq}(r,t)$ represents the equilibrium distribution function to describe the predicted value of redistribution. Δt is the grid size of time, τ is relaxation time, $F_{\alpha }$ is the external force along direction $e_{\alpha }$. Each pixel r has nine nearest neighbors (including itself). The lattice vector $e_{\alpha }$ is the location of each sub-pixel which is defined as:

(5) $$e_{\alpha }=\{\begin{array}{cc} \left(0,0\right) & \alpha =0\\ c\left(\cos\left[\left(\alpha -1\right)\frac{\pi }{2}\right],\sin\left[\left(\alpha -1\right)\frac{\pi }{2}\right]\right) & \alpha =1,\;2,\;3,\;4\\ \sqrt{2}c\left(\cos\left[\left(2\alpha -1\right)\frac{\pi }{4}\right],\sin\left[\left(2\alpha -1\right)\frac{\pi }{4}\right]\right) & \alpha =5,\;6,\;7,\;8 \end{array},$$

During the collision step, $I_{\alpha }$ has a tendency to become $I_{\alpha }^{eq}$. In this step the gray level on each sub-pixel is divided into two parts: One part is to be redistributed, and the other part stays at the original sub-pixel. The collision equation can be expressed as:

(6) $$I_{\alpha }(r,t+\Delta t)-I_{\alpha }(r,t)=\frac{1}{\tau (I)}\left[I_{\alpha }^{eq}(r,t)-I_{\alpha }(r,t)\right],$$

In the streaming step, the gray level on each sub-pixel is updated by the gray level from neighboring sub-pixels. Because $I=\sum_{\alpha }I_{\alpha }$, the gray level of pixels changes. The streaming equation is expressed as:

(7) $$I_{\alpha }(r+e_{\alpha }\Delta t,t+\Delta t)=I_{\alpha }(r,t+\Delta t),$$

In order to improve computing efficiency, we choose the D2Q5 lattice as shown in Figure 3e and apply the Chapman–Enskog expansion method to Equation (4); we get:

(8) $$\frac{\partial I}{\partial t}=div(\frac{2}{5}(\tau (I)-\frac{1}{2})\nabla I)+5F_{\alpha },$$

### 2.4. Lattice Boltzmann Model Driven by DBN

**Figure 4:**
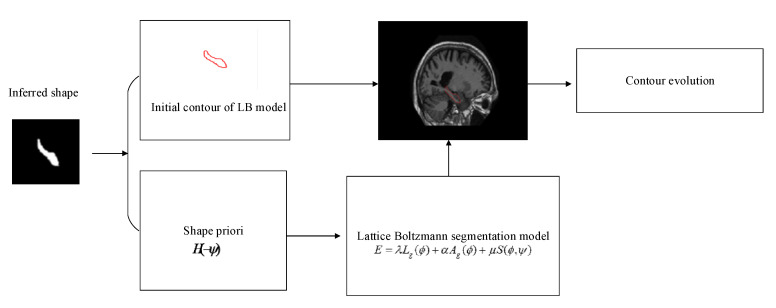
The flowchart of the LB segmentation model.

The flowchart of the LB segmentation model is depicted in Figure 4. The shape inferred from DBN is used not only as part of the external force term of the LB segmentation model, but also to determine the position of the initial contour. We construct an energy function which includes a weighted gradient term, a weighted region term and a shape energy term; these terms are defined as:

(9) $$L_{g}(\phi )=\int_{\Omega }g\delta (\phi )|\nabla \phi |dx,$$

(10) $$A_{g}(\phi )=\int_{\Omega }gH(-\phi )dx,$$

(11) $$S(\phi ,\psi )=\int_{\Omega }(H(-\phi )-H(-\psi ){)}^{2}dx,$$

where ∇ is the gradient operator, $g(x,y)=1/(1+|\nabla G_{\sigma }I(x,y){|}^{2})$ is an edge indicator with $G_{\sigma }$ as the Gaussian kernel and *I* as the image gray level. ψ is the shape prior inferred from the trained DBN, which is defined as:

(12) $$\psi (x,y)=\{\begin{array}{cc} -c, & s(x,y)=1\\ c, & s(x,y)=0 \end{array},$$

where *s* represents the shape mask inferred from the DBN. *H* is the Heaviside function, δ is the Dirac delta function:

(13) $$H_{\varepsilon }(x)=\left\{ \begin{array}{cc} \frac{1}{2\varepsilon }[1+\frac{x}{\varepsilon }+\frac{1}{\pi }\sin(\frac{\pi x}{\varepsilon })], & |x|\leq \varepsilon \\ 1, & x>\varepsilon \\ 0, & x<-\varepsilon \end{array}\right.,$$

(14) $$\delta_{\varepsilon }(x)=\left\{ \begin{array}{cc} \frac{1}{2\varepsilon }(1+\cos(\frac{\pi x}{\varepsilon })), & |x|\leq \varepsilon \\ 0, & |x|>\varepsilon \end{array}\right.,$$

The energy function is:

(15) $$E=\lambda L_{g}(\phi )+\alpha A_{g}(\phi )+\mu S(\phi ,\psi ),$$

where λ>0, μ>0, α∈ℜ. Using the gradient descent flow method to minimize the energy function, we get the following PDE:

(16) $$\frac{\partial \phi }{\partial t}=\lambda \delta_{\varepsilon }(\phi )div(g\frac{\nabla \phi }{\left|\nabla \phi \right|})+\alpha g\delta_{\varepsilon }(\phi )+\mu \delta_{\varepsilon }(\phi )(H_{\varepsilon }(-\phi )-H_{\varepsilon }(-\psi )),$$

Since the distance function |∇ϕ|=1, Equation (16) becomes:

(17) $$\frac{\partial \phi }{\partial t}=\lambda \delta_{\varepsilon }(\phi )div(g\nabla \phi )+F,$$

where:

(18) $$F=\alpha g\delta_{\varepsilon }(\phi )+\mu \delta_{\varepsilon }(\phi )(H_{\varepsilon }(-\phi )-H_{\varepsilon }(-\psi )),$$

Comparing Equations (8) and (16), we get:

(19) $$\tau =\frac{5\lambda g\delta_{\varepsilon }(\phi )+1}{2},$$

(20) $$F_{\alpha }=\frac{\alpha g\delta_{\varepsilon }(\phi )+\mu \delta_{\varepsilon }(\phi )(H_{\varepsilon }(-\phi )-H_{\varepsilon }(-\psi ))}{5},$$

### 2.5. Algorithm

The lattice Boltzmann method in image segmentation can be interpreted as the curve evolution process of the iso-density line (i.e., initial contour) under the action of the internal force (i.e., diffusion) and the external force (i.e., gradient, region, shape prior). The procedure of DBN driven LB method for image segmentation is shown in Algorithm 1 below.

**Algorithm 1.** DBN Driven LB Method for Image Segmentation1: Setting the initial position of evolving curve *C* and defining level set function ϕ as a signed distance function, such as: $\phi (r,0)=\{\begin{array}{c} -c,\\ 0,\\ c, \end{array}\begin{array}{c} r\in C_{in}\\ r\in C\\ r\in C_{out} \end{array}$
where r is the position of one pixel in the image, c>0 is a constant, $C_{in}$ and $C_{out}$ denote the inside and outside region of the evolving curve C, respectively2: Initialize local equilibrium distribution function $\phi_{{}_{\alpha }}^{eq}(r,0)=\frac{1}{5}\phi (r,0)$, compute relaxation parameter τ with Equation (19) 3: Compute the external force term and discretize it with Equation (20)4: Updating the evolving curve and $\phi (r,t)=\sum \phi_{\alpha }^{eq}(r,t)$ after collision and streaming described in Equations (6) and (7), separately5: If the segmentation is not done, jump back to step (2)6: Output the segmentation result

## 3. Experiments

### 3.1. Data Preparation and Experimental Setup

We use a subset of OASIS-1 (http://www.oasis-brains.org/) as the training set of DBN. This dataset was chosen to cover the entire age span of the subjects and to include subjects with different degrees of dementia. A professional radiologist provided manual segmentations of this subset, which are offered publicly [30]. The selected subset consists of 23 right-handed subjects. We sliced the 3D images and their labels along the sagittal direction. In the sagittal direction, each subject has about 20 2D images containing the HC. Each 2D image and its corresponding label is rotated by 60 degrees in steps of 10 degrees. This results in a total of 3220 images for training. Images are rotated so that the model learns the HC located at all possible rotations while training. In order to improve computational efficiency, we manually cropped the images of the training set to a size of 100 × 100. The dataset used for testing is the preliminary release of EADC-ADNI (http://www.hippocampal-protocol.net/) and has manual segmentation over a subset (N = 100). There are 34 patients with MCI, 29 normal control subjects and 37 patients with AD.

The experimental environment is Matlab R2014b installed onto a PC Intel (R) Core (TM) i5 3230M processor with a clock speed of 2.6 GHz and 4 GB of RAM. The parameters in Equation (16) are λ=10, α=0.5, μ=50, based on the training datasets by considering both the stability of the curve evolution and the effectiveness of the shape prior-based guidance. For DBN, there are two hidden layers with 1000 nodes in the first layer and 1000 in the second, and the input and segmentation layers with size 100 *×* 100.

### 3.2. Validation Framework and Evaluation Measures

Firstly, to show the positive effect of shape prior on segmentation results, we subjectively compare the previous LB model and the DRLS method with our method. Secondly, in order to verify that the segmentation method is sensitive to changes of the shape and size of the object, we carry out experiments on the synthetic fishes and HC images. Synthetic fish images have different shapes and sizes. HC images included groups of AD, MCI and NC with different HC volumes. To verify our method and can segment objects with occlusion or missing parts, we carry out experiments on the synthetic ellipse images. Synthetic ellipse images includes partially occluded ellipse and partially missing ellipse. We also compare the segmentation results with ground truth and show the segmentation results of 20 subjects. Thirdly, we use the Dice coefficient as a measure and compare our method with several other methods. Finally, we study the correlation and consistency between several different methods and the ground truth.

## 4. Results

### 4.1. Positive Effect of Shape Prior

**Figure 5:**
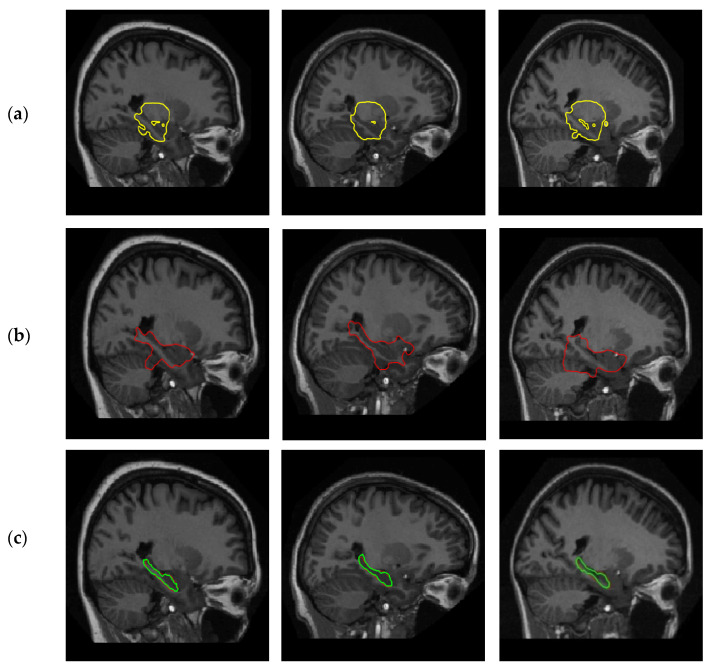
The positive effect of shape prior. (**a**) Results of the method in [33]; (**b**) results of the method in [34]; (**c**) results of our method.

To prove the positive effect of shape prior, we randomly choose three samples from EADC-ADNI and subjectively compare the segmentation results of the proposed method with the previous LB method proposed in [31] and the DRLS method proposed in [32]. The first is an alternative to the Chan–Vese segmentation model; there is no shape term in the segmentation model. The second is a development of the level set method with an additional regularization term in the energy function to avoid shape irregularities and instability during the level set evolution process while eliminating the need for re-initialization. Figure 5 shows the segmentation results of the above two methods and our methods on three randomly selected images: (a) The segmentation result of [31]; (b) the segmentation result of [32]—the segmentation results have serious boundary leakage; and (c) the segmentation result of the proposed method—the green line is the contour draw by expert, the red line is the contour of our method. From Figure 5, we can see that the LB and DRLS methods without prior knowledge cannot segment HC correctly, while the results of our method are close to the ground truth. It is noteworthy that the segmenting time of a slice by LB based methods is less than two seconds, while the segmenting time of the DRLS based method is about 20 s.

### 4.2. Sensitivity to Shape and Size Change

#### 4.2.1. Experiments on Synthetic Images

**Figure 6:**
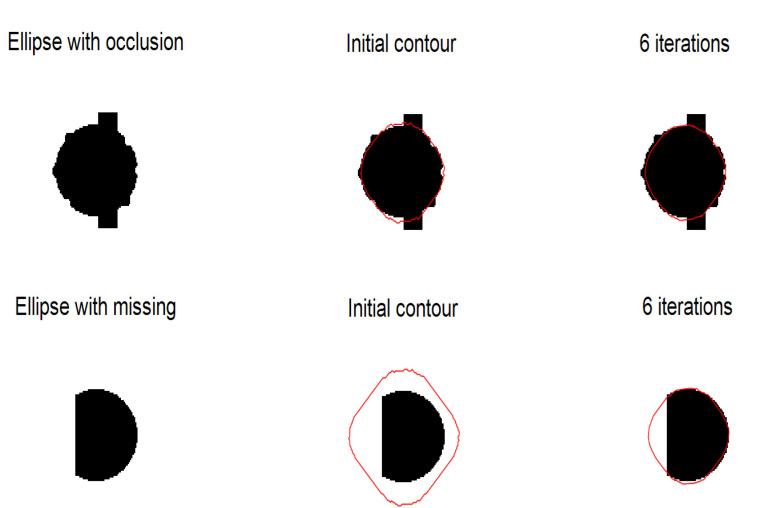
Experiments on ellipse synthetic image. Left column is original image, middle column is initial contour, right column is segmentation results. The first row is experiment on ellipse with occlusion, the second row is experiment on ellipse with missing.

**Figure 7:**
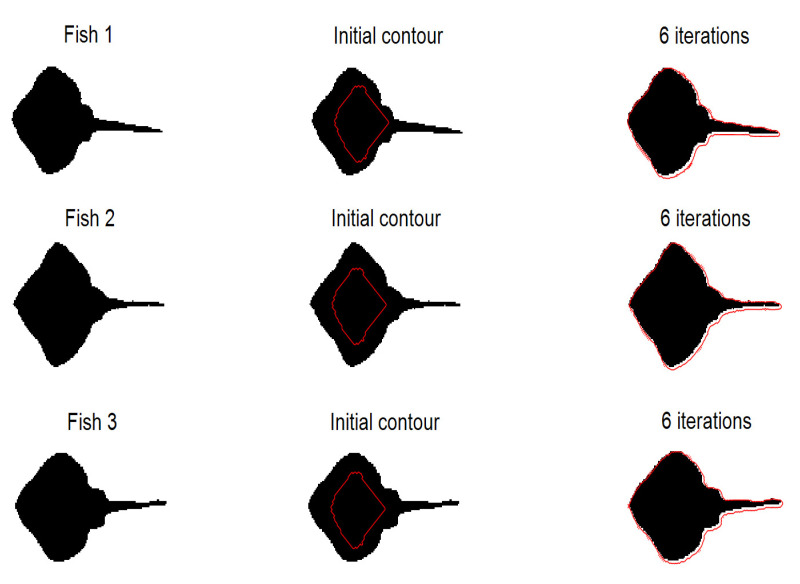
Experiments on three synthetic fishes which are different in size and shape. Left column is original image, middle column is initial contour, right column is segmentation results.

The segmentation results of ellipses are shown in Figure 6. Experiments show that our method can correctly segment objects with partial occlusion and missing parts. The segmentation results of fishes are shown in Figure 7. Experiments show that our method is sensitive to shape and scale variance. It can be found that the initial contour needs only six iterations to reach the edge of the object.

#### 4.2.2. Experiments on Hippocampus Images

**Figure 8:**
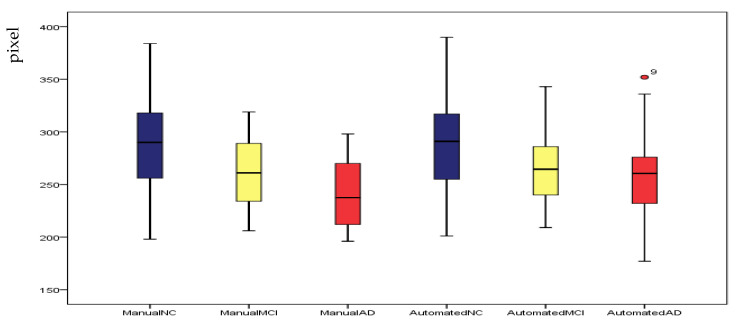
Box plot of our method’s results and ground truths of EADC-ADNI for the three groups.

**Figure 9:**
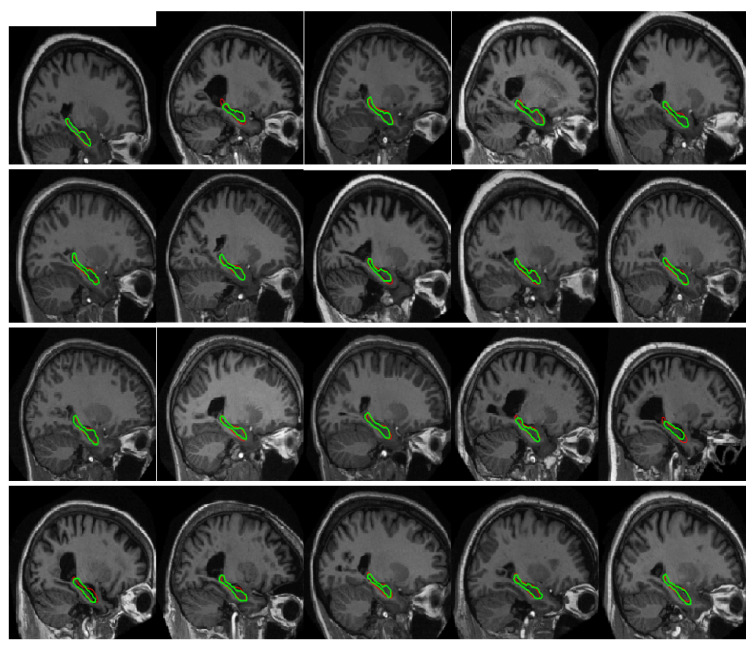
Parts of segmentation results; the green line is the ground truth; the red line is the segmentation results of our method.

To verify the sensitivity of our method to HC structural changes, we segmented images of the AD, MCI and NC groups of EADC-ADNI. We chose the largest HC on the sagittal plane and calculated the average area of three groups, separately. Figure 8 is the boxplot of manual and automated segmentation of the three groups. We find that, although the mean area and area ranges of the three groups after segmentation are different from those of gold standards, the mean values of the three groups are significantly different. This suggests that our method is sensitive to volume changes of HC. Figure 9 shows the segmentation results of some samples, the green line is the ground truth, the red line is the result of our method. From Figure 9 we can find that the results of our method are satisfactory. The segmentation results of samples 2, 8, 14, 15 and 16 are little worse, all of the five samples have severe brain atrophy.

### 4.3. Comparison with Other Methods

In Section 4.1 we proved the LB model without shape prior cannot segment HC correctly. In this part, we compared our method (DBN-LB_jonit) with DBN, PCA-LB_joint [28] and cRBM [29]-LB_jonit. Then we compared our method with state of the art methods including classifier based [33], atlas based [34] and deep learning based [8] methods. The test set used in [8] is the final release of EADC-ADNI with 135 samples. The results of [33] in Table 1 are 50 samples in the preliminary release. The Dice similarity coefficient is used as the evaluation standard, and is defined as:

(21) $$D=\frac{2|A\cap B|}{\left|A|+|B\right|},$$

where set *A* is the estimated volume and *B* is the actual volume.

Table 1 shows the mean and standard deviations of the Dice coefficient from the proposed method and the other six methods on EADC-ADNI. The proposed method achieved an average Dice coefficient of 0.87, which is higher than DBN; the standard deviation of the Dice coefficient of ±0.05 is lower than DBN. This means the combination of DBN and the LB method is effective. Compared with the PCA-LB_joint and cRBM-LB_joint methods, our method has a higher Dice coefficient and lower standard deviation. This means the shape prior inferred from DBN is superior to PCA and cRBM and our method is sensitive to changes in the HC structure between different samples. The Dice coefficient of our method is higher than [8] because that method used FreeSurfer’s outputs as training labels. The multiple random forest classifier method and the multi-atlas method have better results than our method. As the most popular method of brain image segmentation, the multi-atlas method has the lowest standard deviations of the Dice coefficient.

Execution time is an important factor that determines the performance of a segmentation method. PCA-LB_joint, cRBM-LB_joint, DBN-LB_jonit and DRLS can be regarded as the active contour converging to the target boundary. DBN based segmentation is only a part of our proposed method, which is used for rough segmentation. The execution time of the above methods is directly compared in this paper. In our method, the initial contour is very close to the HC edge to be segmented, and the shape prior guides the contour evolution, so the number of convergence iterations is less. Compared with DBN, the shape prior generated from cRBM is not so close to the HC to be segmented, so the cRBM-LB_joint method needs more iterations to converge. The PCA-LB_joint method needs the registration between the shape prior and the HC to be segmented, and the registration process accounts for a large proportion of the total time consumption. DBN-based segmentation is only a part of our proposed method, which is used for rough segmentation, so it does not need an iterative process and takes the shortest time. A comparison of computational cost for the above methods is shown in Table 2. Compared with the traditional methods for solving PDE, the LB method is naturally parallel and has a higher speed. The high computational time of the multi-atlas method is due to the need of registering the target image with various different atlases. In the method proposed in [35], the computational time reported for a volume can take up to eight hours. The time complexity of random forest is related to the depth and width of the decision tree. For convolutional neural networks, the time complexity of each convolution layer is determined by the area of the output characteristic graph, the convolution kernel and the number of input and output channels. We will compare the execution time of the above three methods in the future.

### 4.4. Correlation and Consistency

**Figure 10:**
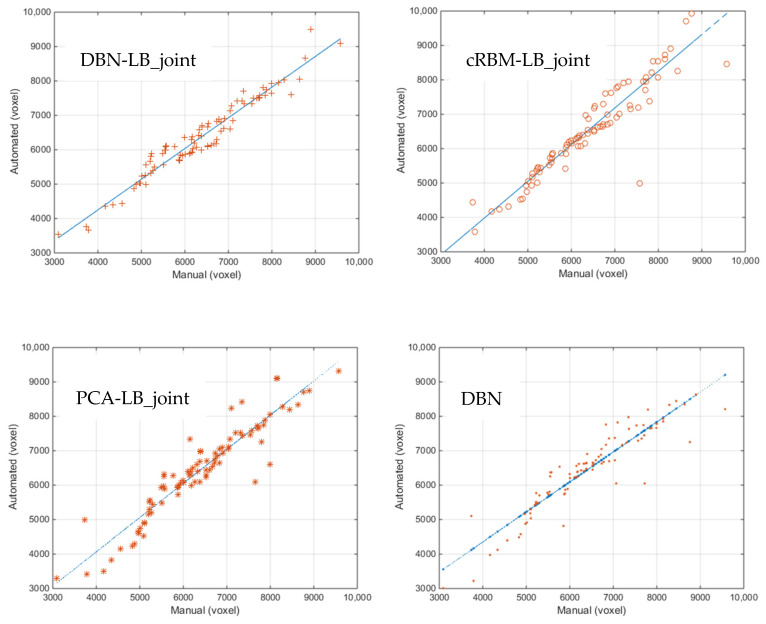
Manual and automated volumes for the DBN-LB_joint, cRBM-LB_joint, PCA-LB_joint and DBN methods.

Figure 10 shows scatter plots of the volumes estimated by DBN-LB_joint, cRBM-LB_jiont, PCA-LB_joint and DBN versus the manually measured volumes. We observe a clear correlation between the four methods and the ground truth, together with a number of outliers. The volumetric intraclass correlation coefficients (ICCs) of the four methods are 0.97, 0.94, 0.93 and 0.92 respectively. The results of the four methods are all statistically significant. Compared with other three methods, our method has the highest ICC and the fewest outliers.

**Figure 11:**
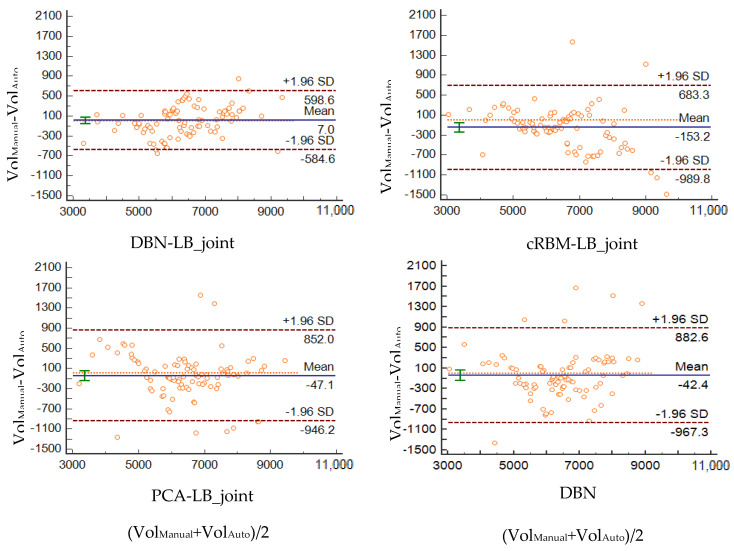
Bland–Altman plots for the EADC-ADNI dataset showing graphically the agreement between the manually segmented volumes and the volumes segmented by means of DBN-LB_joint, cRBM-LB_joint, PCA-LB_joint and DBN method.

Furthermore, the agreement between the automatically and manually segmented volumes was studied with the use of Bland–Altman analysis (Figure 11). cRBM-LB_joint, PCA-LB_joint and DBN methods all present an overestimation bias. Furthermore, PCA-LB_joint and DBN show a light tendency to underestimate small volumes; the cRBM-LB_joint method shows a light tendency to overestimate the large volumes. The DBN-LB_joint method has a much lower bias when compared to the other three methods. This indicates that the volumes calculated by means of the DBN-LB_joint method are closer to the manually segmented ones.

## 5. Discussion

DRLS and LB models without shape prior cannot segment HC correctly because its boundaries present rich, poor and missing gradient regions. Our model uses both the global constraint of shape and the local information of the target image. Global constraint ensures our model can deal with different types of images including partial occlusion, missing parts, weak boundaries. Local information ensures our model has the ability to deal with objects with shape and scale variance.

Experiments on synthetic image and an HC image show our model is sensitive to shape and scale change and can segment objects with partial occlusion and missing parts. While the effect of this method in the segmentation of HC in patients with severe brain atrophy is little worse, the statistical results are satisfactory. We are confident of solving this problem by increasing the number of hidden layers and selecting training samples reasonably.

That the segmentation accuracy of the DBN-LB_joint is higher than using DBN and the LB method independently means that this combination is effective. That the segmentation accuracy of our method is higher than PCA-LB_joint and cRBM-LB_joint means the shape prior generated from DBN is superior to those generated from PCA and cRBM. The reason for this is that PCA methods extract several principal components from a set of training images as a shape prior, which inevitably loses useful information. The cRBM lacks the ability to receive feedback from a higher layer to a lower layer and might generate a sub-optimal shape model. Compared with cRBM, DBN can capture more global information of the HC shape. The segmentation results of our method are worse than the atlas based method because the latter has mature template.

## 6. Conclusions

We proposed a DBN-driven LB model for HC segmentation. Compared with the statistical shape prior, the shape prior inferred from the DBN is patient specific. Our method does not need train label alignment, shape prior or target object registration. Our method is sensitive to the changes of the HC structure and has good correlation and consistency with the results of manual segmentation. While satisfactory segmentation results were achieved in this paper, there are several directions deserving further study. (1) We will study a method that can automatically locate the HC to generate the training set of DBN; (2) the number of layers and units in the hidden layer of DBN are determined manually. We will study an optimization method that can automatically determine the above parameters. (3) Considering the natural parallelism of the LB method, we plan to apply it directly to 3D HC segmentation in the future. (4) Furthermore, we plan to use the generative model such as RBM, DBN or DBM to get the occlusion sensitive map of organs, which plays an important role in medical image analysis.

## Figures and Tables

**Table 1:** Comparison results using mean Dice’s coefficient and standard deviations of the Dice coefficient.

Method	Diceμ+δ	Method Description
DBN	0.84 ± 0.07	DBN separately
PCA-LB_joint	0.84 ± 0.06	PCA driven LB
cRBM-LB_joint	0.85 ± 0.06	RBM driven LB
DBN-LB_jonit	0.87 ± 0.05	DBN driven LB
multiple Random Forest classifier [33]	0.87 ± 0.03	multiple Random Forest classifier
multi-atlas [34]	0.88 ± 0.02	integrating label propagation and random forests method
Deep learning [8]	0.85	deep convolutional neural networks

**Table 2:** Average convergence time for DBN, PCA-LB_joint, cRBM-LB_joint, DBN-LB_jonit and DRLS methods on testing datasets.

Method	Time (s/slice)	The Number of Iterations
DBN	0.878	
PCA-LB_joint	6.035	11
cRBM-LB_joint	3.587	9
DBN-LB_jonit	2.220	6
DRLS	21.984	210
